# Detection of CD133 (prominin-1) in a human hepatoblastoma cell line (HuH-6 clone 5)

**DOI:** 10.1002/jemt.22237

**Published:** 2013-05-27

**Authors:** Masumi Akita, Kayoko Tanaka, Noriko Murai, Sachiko Matsumoto, Keiko Fujita, Takashi Takaki, Hidetoshi Nishiyama

**Affiliations:** 1 Division of Morphological Science, Biomedical Research Center, Saitama Medical University38 Moroyama, Iruma-gun, Saitama, 350–0495, Japan; 2 Department of Anatomy, Saitama Medical University38 Moroyama, Iruma-gun, Saitama, 350–0495, Japan; 3 Advanced Technology DivisionJEOL Ltd., Akishima, Tokyo, 196–8558, Japan

**Keywords:** cancer stem cell, ASEM, nanogold, filopodia, lamellipodia, methyl-β-cyclodextrin

## Abstract

We examined CD133 distribution in a human hepatoblastoma cell line (HuH-6 clone 5). We directly observed the cultured cells on a pressure-resistant thin film (silicon nitride thin film) in a buffer solution by using the newly developed atmospheric scanning electron microscope (ASEM), which features an open sample dish with a silicon nitride thin film window at its base, through which the scanning electron microscope beam scans samples in solution, from below. The ASEM enabled observation of the ventral cell surface, which could not be observed using standard SEM. However, observation of the dorsal cell surface was difficult with the ASEM. Therefore, we developed a new method to observe the dorsal side of cells by using Aclar® plastic film. In this method, cells are cultured on Aclar plastic film and the dorsal side of cells is in contact with the thin silicon nitride film of the ASEM dish. A preliminary study using the ASEM showed that CD133 was mainly localized in membrane ruffles in the peripheral regions of the cell. Standard transmission electron microscopy and scanning electron microscopy revealed that CD133 was preferentially concentrated in a complex structure comprising filopodia and the leading edge of lamellipodia. We also observed co-localization of CD133 with F-actin. An antibody against CD133 decreased cell migration. Methyl-β-cyclodextrin treatment decreased cell adhesion as well as lamellipodium and filopodium formation. A decrease in the cholesterol level may perturb CD133 membrane localization. The results suggest that CD133 membrane localization plays a role in tumor cell adhesion and migration.

## INTRODUCTION

Recently, the hypothesis of cancer stem cells (CSCs) was proposed to explain the origin of cancer cells. By definition, CSCs are a small fraction of tumor cells with the capacity of both self-renewal and unlimited slow proliferation. They are often resistant to chemotherapy and radiation and are thus responsible for continuously supplying new cancer cells (Zhao et al., 36). CSCs exhibit specific cell membrane markers. In human hepatocellular carcinoma (HCC) and HCC cell lines, specifically CD133+ cells, and not CD133− cells, have the ability to self-renew, produce differentiated progenies, and form tumors (Ma et al., 11; Suetsugu et al., 28). CD133 has also been used as a marker for CSCs in many different solid tumors, including colon (O'Brien et al., 20; Ricci-Vitiani et al., 24), brain (Liu et al., 9; Singh et al., 27), skin (Monzani et al., 17), pancreatic (Olempska et al., 21), liver (Hayashi et al., 6; Yin et al., 35), and prostate (Collins et al., 1) tumors. However, Quintana et al. (22) and Shackleton et al. (26) showed that tumors that arose from both CD133− and CD133+ cells sorted from an original melanoma re-established the original ratios of CD133− and CD133+ cells. This experiment indicates that individual cancer cells can recapitulate the marker heterogeneity of the tumors from which they are derived.

Recent evidence has revealed that CD133 is widely expressed in many organs (Shmelkov et al., 37). CD133, also known as prominin-1 in humans and rodents, was first isolated and cloned in 1997 (Miraglia et al., 16; Weigmann et al., 33). The CD133 antigen (AC133) is a 97-kDa glycoprotein with five transmembrane domains. AC133 is a glycosylated epitope of the CD133 protein and was initially found to be associated with embryonic stem cells (King et al., 8). The expression of CD133 in various embryonic and adult tissues has been studied by examining the presence of prominin mRNA as well as AC133 (human) and 13A4 (mouse) immunoreactivities. CD133 expression is not restricted to neuroepithelial and hematopoietic stem/progenitor cells in which it was originally observed, but extends to several epithelial and non-epithelial cell types. The biological function of CD133 remains largely unknown. No natural ligand of CD133 has yet been identified. Recently, LS-7 (amino acid sequence, LQNAPRS), a specific binding peptide targeting mouse CD133, was screened and identified for the first time by using the phage-display peptide library technology (Sun et al., 30). Yi et al. (34) reported that CD133 and the interleukin-6 receptor are co-expressed in lung CSCs. However, at the subcellular level, the localization of CD133 remains unknown.

In this study, we examined the distribution of CD133 in a human hepatoblastoma cell line (HuH-6 clone 5). We directly observed the cultured cells in a buffer solution by using the newly developed atmospheric scanning electron microscope (ASEM). This microscope features an open sample dish with a silicon nitride thin film window at its base, through which the scanning electron microscopy (SEM) beam scans samples in solution, from below (Nishiyama et al., 19; Sato et al., 25; Suga et al., 29). Standard electron microscopy has sub-nanometer or nanometer resolution, but the samples must be observed under vacuum. This requires time-consuming pretreatments and is inappropriate for quick diagnoses. With its high resolution and in-solution observation capabilities, the ASEM is well suited to the observation of thin cultured cells. A preliminary study using the ASEM showed that CD133 was localized in the membrane ruffles found at the peripheral regions of the cell. Standard transmission electron microscopy (TEM) and SEM revealed that CD133 was preferentially concentrated in a complex structure comprising filopodia and the leading edge of lamellipodia. The protrusive structures at the leading edge of a motile cell are called lamellipodia and filopodia. A lamellipodium is a thin sheet-like protrusion that is filled with a branched network of actin. In contrast, filopodia are thin, finger-like structures that are filled with tight parallel bundles of filamentous F-actin. Cell migration is an extensively studied process that depends on several dynamic actin assemblies (Mattila and Lappalainen, 14). The present study revealed the co-localization of CD133 with F-actin. Furthermore, an antibody against CD133 decreased cell migration, strongly suggesting that CD133 is involved in tumor cell migration.

## Materials and methods

### Cell Culture

Human hepatoblastoma cells (HuH-6 clone 5, well-differentiated type, JCRB0401) were purchased from the Health Science Research Resources Bank (Osaka, Japan). The cells were cultured on a 100-nm thick silicon nitride film (formed by chemical vapor deposition and wet etching) and Aclar® plastic film (Nisshin EM, Tokyo, Japan) in Dulbecco's modified Eagle's medium (DMEM) supplemented with 10% fetal bovine serum (FBS) and 50 µg/mL gentamicin, according to a method previously reported by Hayashi et al. (6). The Aclar® plastic films were sterilized by immersion in 70% ethanol for 20 min. The films were then rinsed twice in phosphate-buffered saline (PBS) and placed on the bottom of plastic culture dishes (35 mm).

### ASEM

Affinity labeling with gold conjugates was performed using a modification of a previously published method (Nishiyama et al., 19), and images were acquired using the newly developed ASEM. Cells were cultured on a thin silicon nitride film and Aclar® plastic film and fixed with 4% paraformaldehyde in PBS at room temperature for 10 min. To detect CD133 on the cell surface, cells were incubated with 1% skim milk/PBS for 30 min, after which CD133 antibody (Santa Cruz Biotechnology, CA) was added with further incubation for 1 h. Colloidal gold (EM goat F(ab)_2_ anti-rabbit IgG:10 nm gold; BB International, UK) was used as the secondary antibody. Alexa Fluor 488- and nanogold (1.4 nm)-conjugated goat anti-rabbit IgG (Nanoprobes, NY) were also used as secondary antibodies; the incubation period was 30 min. The nanogold signal was enhanced using GoldEnhance EM (Nanoprobes, NY, USA) at room temperature for 3 min. Before observation under the ASEM, the sample was further fixed with 1% glutaraldehyde in PBS at room temperature for 10 min. After 1% glutaraldehyde fixation, the samples were stained with phosphotungstic acid to achieve high contrast during imaging with the ASEM. The labeled cells in 10 mg/mL glucose (a radical scavenger) were directly observed under the ASEM. The ASEM can be used to observe labeled cells in an open medium through the thin silicon nitride film. The ASEM can also observe cells cultured on an Aclar® plastic film through the thin silicon nitride film.

### Immunohistochemistry for Light Microscopy

Cultured cells were fixed in 4% paraformaldehyde/PBS and immunohistochemistry for CD133 was performed using streptavidin/peroxidase. The specimens were treated with 0.3% H_2_O_2_ in methanol to block endogenous peroxidase activity and were subsequently incubated with a primary CD133 antibody (Santa Cruz Biotechnology, CA). A biotinylated anti-rabbit IgG was added as a secondary antibody. A horseradish peroxidase-labeled streptomycin–avidin complex was then used to detect the secondary antibody. The antibody complex was detected by staining with the chromogen 3,3′-diaminobenzidine. Fluorescein isothiocyanate (FITC)-conjugated goat anti-rabbit IgG (Cappel; ICN Pharmaceuticals, OH) was also used as a secondary antibody and the cultured cells were stained with rhodamine-phalloidin (Invitrogen Corp., CA) to detect the presence of F-actin.

Alexa Fluor 488- and nanogold (1.4 nm)-conjugated goat anti-rabbit IgG (Nanoprobes) were also used as secondary antibodies. The nanogold signal was enhanced using GoldEnhance EM (Nanoprobes) at room temperature for 20 min.

### Migration Assay

Cells were cultured to near confluence at 37°C in 35-mm culture dishes containing DMEM supplemented with 10% FBS. A linear scratch wound was made on the surface of the cultured cells using a sterile blade, according to the scratch wound healing assay (Liu et al., 10). Then, the cells on one side of the scratch wound were completely scraped off using a plastic scraper, and the remaining cells were washed with Hank's buffered salt solution. The cultures were treated without (control group) or with two concentrations of CD133 antibody (1 and 10 μg/mL treatment groups, four dishes per concentration) in fresh culture medium at 37°C in 5% CO_2_ for 24 h. Migrating cells were observed under an inverted microscope (BZ9000; Keyence, Osaka, Japan). The distance migrated by the cells was measured from at least 10 images obtained from each dish and has been expressed as mean ± SD values. Statistical differences were evaluated using the Student's *t*-test. *P* < 0.01 was considered statistically significant.

### Detergent Extraction of Cells

The cells were treated with 5 mM methyl-β-cyclodextrin (MβCD) (Sigma-Aldrich, Tokyo, Japan) for 1 h and fixed with 4% paraformaldehyde in PBS at room temperature for 10 min. The methods used for the immunohistochemical analysis of CD133 were the same as those described above.

### TEM

The cells were fixed in 0.1 M phosphate buffer (pH 7.2) containing 2.5% glutaraldehyde for 1 h, followed by fixation in 0.1 M phosphate buffer (pH 7.2) containing 1% OsO_4_ for another hour. The specimens were dehydrated in graded ethanol, embedded in epoxy resin, cut into ultrathin sections, and stained with uranyl acetate and lead citrate. The stained ultrathin sections were observed under a TEM (JEM-1010; Tokyo, Japan).

### SEM

For standard SEM, the samples were fixed in 0.1 M phosphate buffer (pH 7.2) containing 2.5% glutaraldehyde for 1 h and subsequently fixed in 0.1 M phosphate buffer (pH 7.2) containing 1% OsO_4_ for 1 h, dehydrated in graded ethanol, and critical-point air-dried after treatment with isoamyl acetate. The samples were sputter coated with OsO_4_ and observed under a SEM (Hitachi, S-4800; Tokyo, Japan).

## RESULTS

### Surface Localization and Distribution of CD133 and F-actin by Using Light Microscopy and ASEM

**Figure 1:**
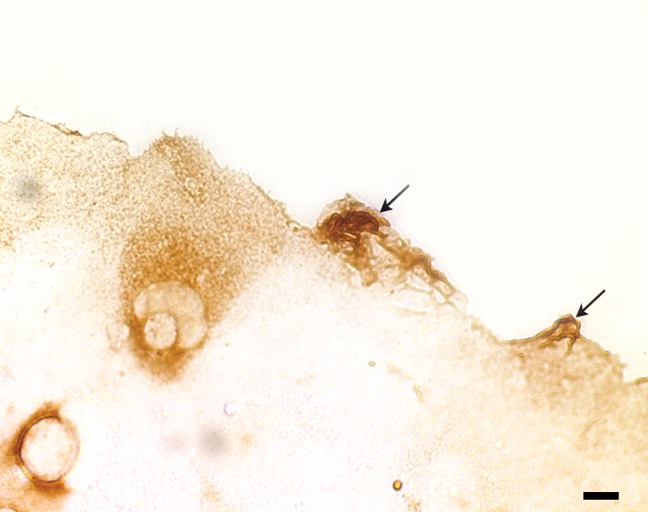
Localization of CD133 by light microscopy. Peripheral regions of the cell are strongly positive (arrows). Scale bar, 10 μm. [Color figure can be viewed in the online issue, which is available at wileyonlinelibrary.com.]

**Figure 2:**
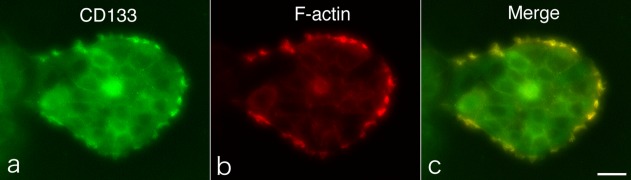
Co-localization of CD133 and F-actin. a : Localization of CD133 by using FITC-conjugated secondary antibody. b: Localization of F-actin by using rhodamine-phalloidin. c: Merged image. CD133 and F-actin are co-localized at the peripheral regions of the cell. Scale bar, 40 μm. [Color figure can be viewed in the online issue, which is available at wileyonlinelibrary.com.]

**Figure 3:**
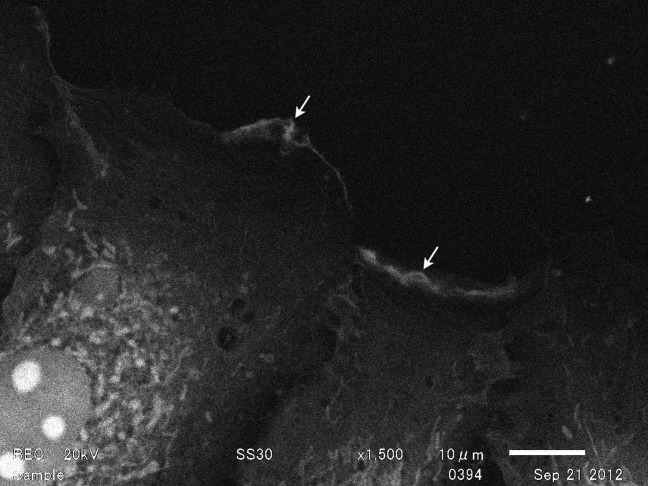
Localization of CD133 by using the ASEM. CD133 is also localized in membrane ruffles seen at the peripheral regions of the cell. Sites with high concentrations of colloidal gold are distinguishable (arrows), although the nanoparticles are not visible.

**Figure 4:**
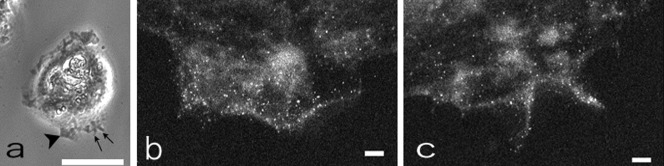
Localization of CD133 using phase contrast microscopy and ASEM. a: Phase contrast micrograph showing a lamellipodium (arrowhead) and filopodia (arrows). Scale bar, 40 µm. b, c: Localization of CD133 by using ASEM. Cell-surface CD133 was labeled with nanogold, followed by gold enhancement. The clustered particles form a white spot approximately 50 nm in size, mainly in the lamellipodium and filopodia. Scale bar, 1 µm. b: Enlarged ASEM image of the lamellipodium indicated by an arrowhead in Figure 4a. c: Enlarged ASEM image of the filopodia indicated by arrows in Figure 4a.

**Figure 5:**
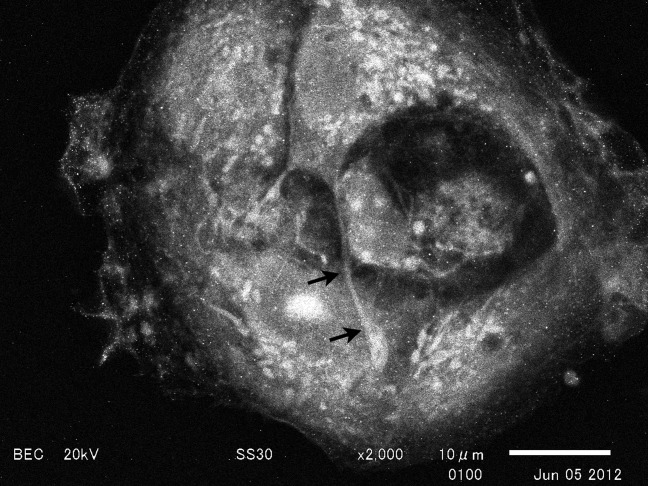
ASEM image of a cell after counterstaining with phosphotungstic acid. The counterstain is effective for visualizing greater details of cells. Ventral cell surfaces are observed. The arrows indicate a long cell process.

**Figure 6:**
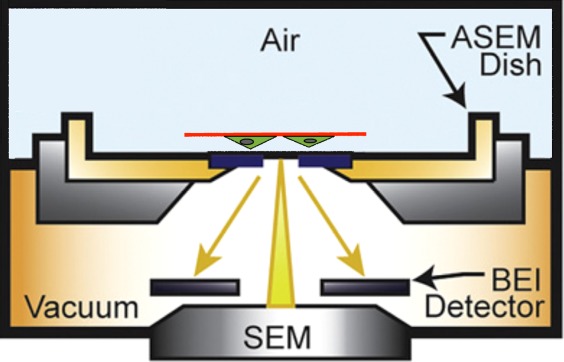
ASEM observation of cells through Aclar® plastic film. The two green triangles indicate cultured cells on Aclar® plastic film (red). The Aclar® plastic film was set upside down. [Color figure can be viewed in the online issue, which is available at wileyonlinelibrary.com.]

**Figure 7:**
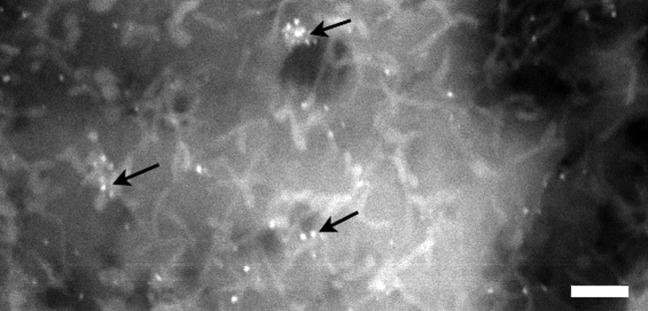
Observation of the dorsal surface of a cell with the ASEM. Cell-surface CD133 was labeled with nanogold, followed by gold enhancement. CD133 is mainly detected in microvillus-like plasma membrane protrusions (microspikes) on the dorsal surface of the cell (arrows). Scale bar, 1 µm.

Under a light microscope, strong positive results were seen for CD133 at the cell periphery (Fig. 1), where it co-localized with F-actin (Figs. 2a–2c). Preliminary examination using ASEM also showed that CD133 was localized in membrane ruffles, which occur at the peripheral regions of the cell (Fig. 3). The ASEM enables direct observation of subcellular structures, unlike phase contrast microscopy. Lamellipodia, characteristic features at the leading edge of the cell, were observed. Filopodia were observed as slender cytoplasmic projections that extended beyond the leading edge of lamellipodia. We labeled cell-surface CD133 with nanogold, followed by gold enhancement. The clustered particles formed a white spot, up to 50 nm in size, mainly in the membrane ruffles formed by lamellipodia and filopodia (Fig. 4). Counterstaining with phosphotungstic acid revealed the details of the cells, and ASEM detected details of the ventral cell surface (Fig. 5). When the Aclar® plastic film was set upside down, the dorsal surface of the cell could be observed. CD133 was mainly localized in microvillus-like plasma membrane protrusions (microspikes) on the dorsal surface of the cells Figs.(6 and 7).

### Localization of CD133 by Using Standard SEM and TEM

**Figure 8:**
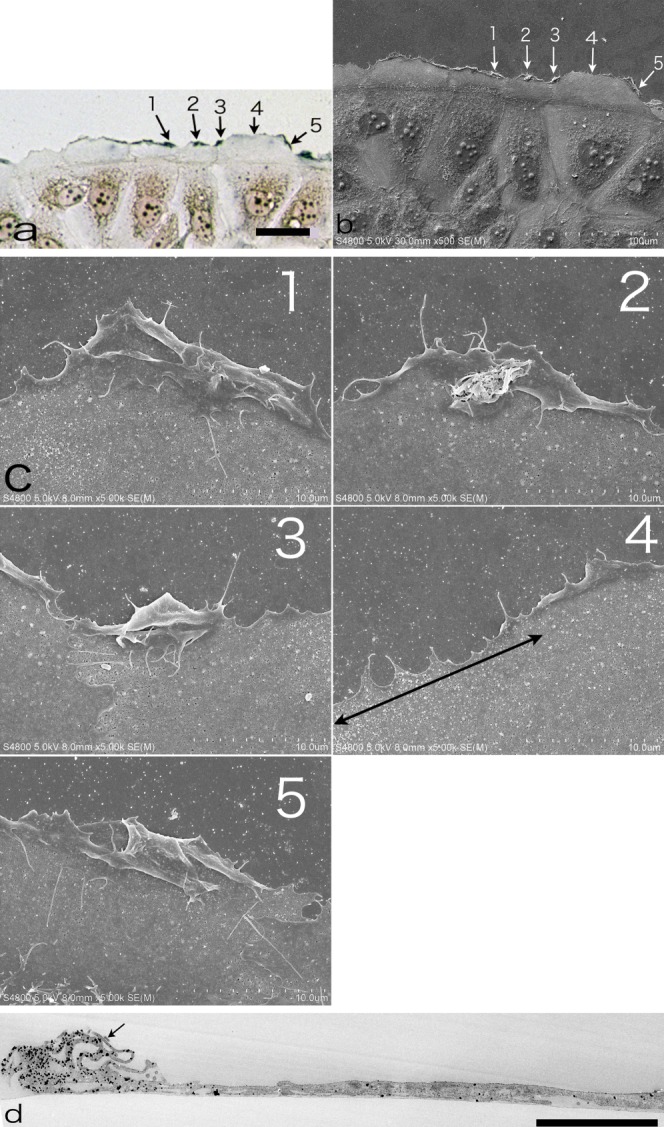
Immunohistochemistry of CD133 viewed using light microscopy, standard SEM, and TEM. a: Light microscopy. CD133 is strongly detected at the peripheral regions of cells (arrow nos. 1, 2, 3, and 5). Nanogold labeling was followed by gold enhancement at room temperature for 20 min. Arrow no. 4 indicates a negative site. Scale bar, 50 µm. b: Standard SEM image of the sample shown in (a). The peripheral region of cells (arrow nos. 1, 2, 3, and 5) coincides with the positive sites observed by light microscopy (see a). Arrow no. 4 indicates a negative site. c: Enlarged standard SEM images of the positive sites shown in (b) (arrow nos. 1, 2, 3, 4, and 5). The positive sites (arrow nos. 1, 2, 3, and 5) are composed of a complex structure of filopodia and lamellipodia. Arrow no. 4 indicates a negative site. In particular, the negative site (black line with arrows at both ends) is a simple structure made up of filopodia and lamellipodia. d: TEM image. CD133 is preferentially concentrated in the complex structure of filopodia and the leading edge of lamellipodia (arrow). The clustered particles form black spots. Scale bar, 5 µm. [Color figure can be viewed in the online issue, which is available at wileyonlinelibrary.com.]

CD133 was strongly detected at the peripheral regions of the cell (Fig.8a). Examination of the CD133-positive sites using standard SEM revealed that they coincided with filopodia and lamellipodia (Figs.8b and 8c).

TEM revealed that CD133 was preferentially concentrated in a complex structure formed by filopodia and lamellipodia (Fig.8d).

### Effect of CD133 Antibody on Cell Migration

**Figure 9:**
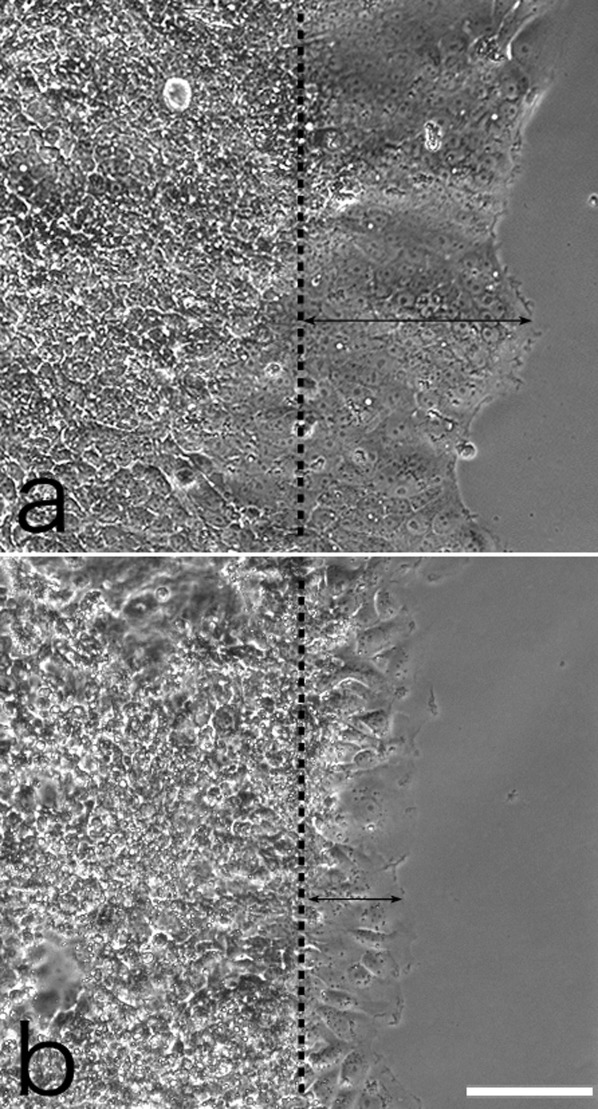
Inhibitory effect of CD133 antibody on cell migration. A migration assay was performed using the scratch wound healing assay and cells were viewed under an inverted optical microscope. a: Control. b: CD133 antibody treatment (10 μg/mL). Black lines with arrows at both ends indicate the migration distance of cells into the scraped areas. Dashed lines show the initial leading edges. Scale bar, 100 µm.

As shown in Figure 9, cell migration decreased after treatment with a CD133 antibody (10 μg/mL) for 24 h. The distance of cell migration from the leading edge was significantly shortened by the CD133 antibody treatment (control group, 187.6 ± 16.0 μm; CD133 treatment group, 96.9 ± 8.2 μm, *P* < 0.01). However, the cell migration distance was not significantly affected at the lower antibody concentration (1 μg/mL).

### Effect of MβCD on Cell–Cell Contact and Morphology

MβCD treatment induced morphological changes in the cells. Cell–cell contact and cell spreading reduced, and many of the cells exhibited an oval shape (Figs. a–d). At the ultrastructural level, MβCD treatment decreased cell–cell contact (Fig. 11), and the CD133-positive cells acquired a round shape. Although lamellipodia and filopodia formation reduced on MβCD treatment, CD133 immunoreactivity was preserved (Figs.12 and 13).

**Figure 10:**
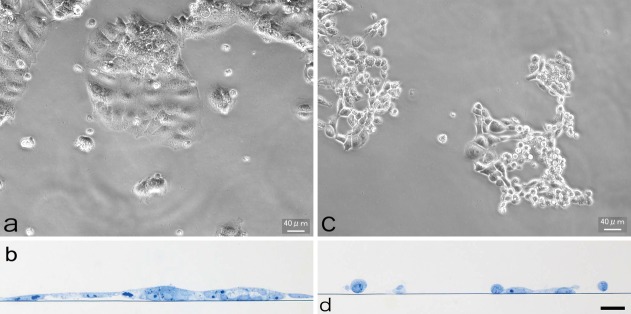
Effect of methyl-β-cyclodextrin (MβCD) on cells. a, b: Control. Cells are well spread and cell–cell adhesion is well developed. a: Phase contrast microscopy; b: thin section stained with toluidine blue. c, d: MβCD treatment induces morphological changes in cells. Cell–cell adhesion is diminished and cells acquire a round shape. Scale bar, 40 µm (for b and d). [Color figure can be viewed in the online issue, which is available at wileyonlinelibrary.com.]

**Figure 11:**
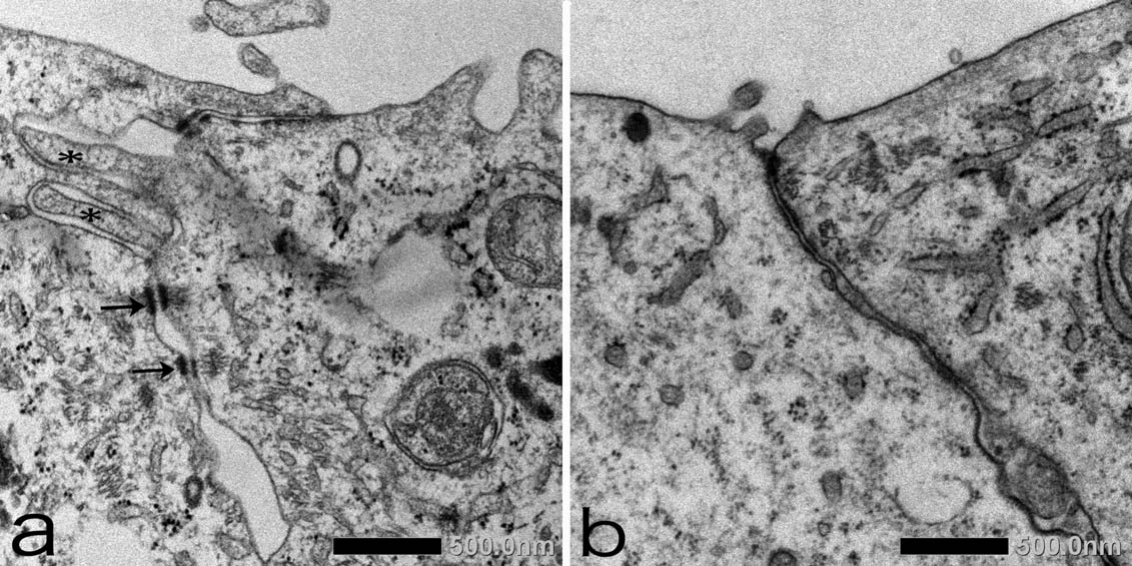
TEM analysis of cells after MβCD treatment. a: Control. Desmosomes (arrows) and intercellular digitations (asterisks) are well developed. b: MβCD treatment. Cell–cell adhesion is diminished. Desmosomes and intercellular digitations are not observed.

**Figure 12:**
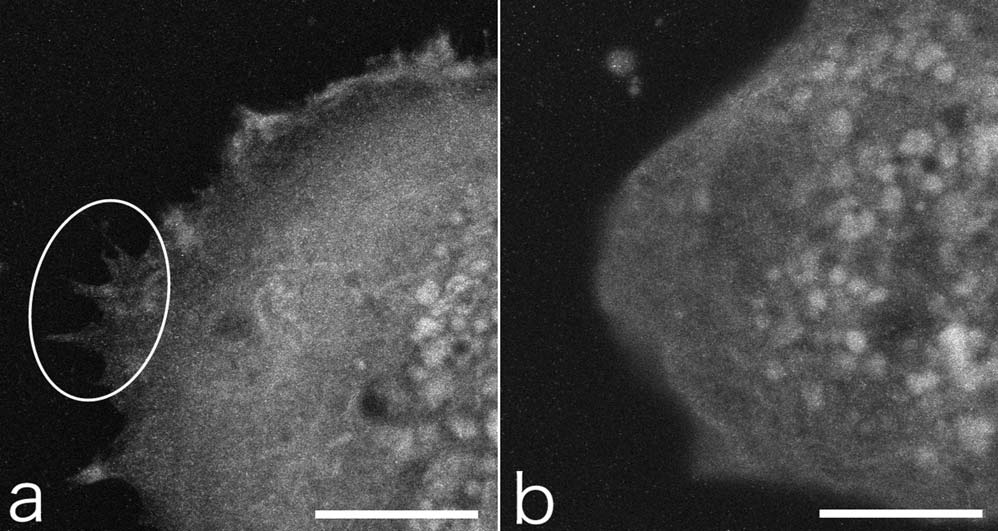
ASEM analysis of cells after MβCD treatment. a: Control. Lamellipodia and filopodia (encircled by a white border) are clearly observed. Scale bar, 10 µm. b: MβCD treatment decreases lamellipodium and filopodium formation. Scale bar, 10 µm.

**Figure 13:**
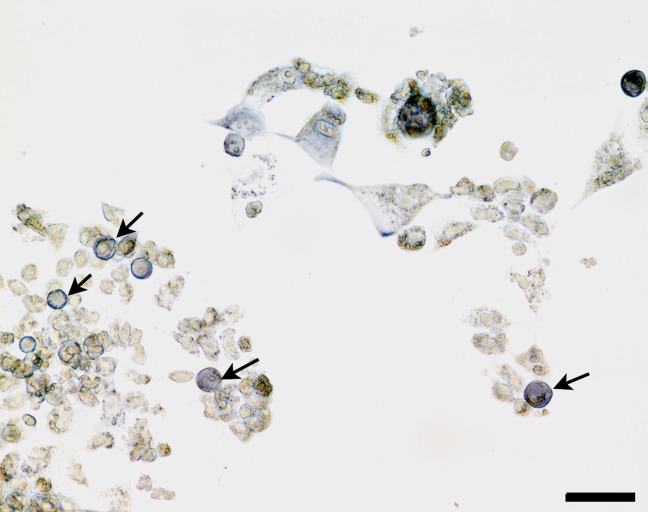
Localization of CD133 after MβCD treatment. Many CD133-positive cells (arrows) acquire a round shape and retain CD133-positive immunoreactivity. Scale bar, 40 µm. [Color figure can be viewed in the online issue, which is available at wileyonlinelibrary.com.]

## DISCUSSION

The new ASEM builds on thin-film technology and can be used to visualize samples in an open solution (Nishiyama et al., 19). The open 35-mm dish of the ASEM can achieve high-throughput staining and/or labeling (Nishiyama et al., 19). In conventional electron microscopy, samples are imaged in vacuum. Therefore, the sample has to be dehydrated and dried; this is a time-consuming and labor-intensive process. On the other hand, ASEM allows the observation of samples in aqueous solution; thus, sample preparation time can be significantly shortened (Nishiyama et al., 19). Because all processes are performed in aqueous solution, antigenicity is well preserved (Maruyama et al., 13). Consequently, the throughput of observation can be significantly improved compared with that for conventional electron microscopy. This is especially effective for rapid screening of samples. An optical microscope observes samples from above, and a pressure-resistant silicon nitride thin film window in the base of the detachable dish allows the inverted SEM to scan samples from below. Although standard SEM revealed the fine structures of the dorsal cell surface, observation of the ventral cell surface was not possible with this microscope. The ASEM, on the other hand, makes it possible to observe the ventral cell surface, as shown in the present study. However, observation of the dorsal cell surface, especially in thick and stratified cells, was difficult because of electron scattering in the sample. This is a limitation of the ASEM. However, we developed a new method for observing the dorsal cell surface by using Aclar® plastic films. To date, little attention has been paid to the observation of the ventral cell surface, despite its importance in understanding cell migration and tumor cell invasion. This new method could be applied to analyze cellular invasion and neurite outgrowth by using cell culture insert systems, which are suitable for a variety of cell-based assays that require cells to have apical and basolateral access to media.

In the epithelial cells of tissues and cell lines expressing endogenous or transfected CD133, CD133 is localized exclusively on microvilli and is not detected on the planar regions of the apical domain (Collins et al., 1; Corbeil et al., 2–3). Because tight junctions prevent the lateral diffusion of CD133 into the lateral plasma membrane, CD133 maintains its apical localization (Corbeil et al., 2–4; Dragsten et al., 5). The mechanism underlying the microvillus-specific localization is unclear. In non-epithelial cells, such as hematopoietic progenitor cells, CD133 is also enriched in plasma membrane protrusions (Corbeil et al., 3). In rod photoreceptor cells, CD133 is concentrated in the plasma membrane evaginations present at the base of the outer segment (Maw et al., 15). CD133 is also concentrated in microspikes, filopodia, and the leading edge of lamellipodia in fibroblasts expressing transfected CD133 (Collins et al., 1; Corbeil et al., 3). In the present study, we showed that CD133 was preferentially concentrated in a complex structure comprising filopodia and the leading edge of lamellipodia. Abundant filopodia are also considered a characteristic of invasive cancer cells (Vignjevic et al., 32). The present study revealed the co-localization of CD133 with F-actin. Furthermore, an antibody against CD133 decreased cell migration, strongly suggesting that CD133 is involved in tumor cell migration.

CD133 is a cholesterol-binding protein that is associated with a characteristic cholesterol-based membrane lipid microdomain (often referred to as lipid rafts) (Röper et al., 23). MβCD is used to selectively extract membrane cholesterol and disrupt lipid rafts (Ilangumaran and Hoessli, 7), and cholesterol depletion leads to the disorganization of the lipid raft structure (Murai, 18). Disruption of lipid rafts using MβCD abolishes lamellipodia formation and inhibits the chemotactic migration of mammary cancer cells (MCF-7) (Mañes et al., 12). Our present study also confirmed that MβCD abolishes lamellipodia and filopodia formation. MβCD diminished cell adhesion by decreasing desmosomes and intercellular digitations. Although the cells retained CD133 after MβCD treatment, a decrease in the cholesterol levels may perturb the regulated membrane localization of CD133. Vasioukhin et al. (31) reported that inhibition of filopodia formation by the expression of a dominant-negative form of the small GTPase CDC42 impaired cell spreading. Further studies are required to understand the biological function of CD133 in lamellipodia and filopodia.
